# Parvovirus B19 and Parvovirus 4 infections among healthy blood donors; A prevalence report from Iran

**DOI:** 10.1016/j.idcr.2024.e02055

**Published:** 2024-08-05

**Authors:** Mohammadmahdi Sabahi, Mehrdad Mosadegh, Azin Kazemi, Razieh Amini, Shahab Mahmoudvand, Mojtaba Hedayat Yaghoubi, Mohammad Masoud Maleki, Zahra Sanaei, Farid Azizi Jalilian

**Affiliations:** a Department of Neurological Surgery, Pauline Braathen Neurological Center, Cleveland Clinic Florida, Weston, FL, USA; b Department of Pathobiology, School of Public Health, Tehran University of Medical Sciences, Tehran, Islamic Republic of Iran; c Student Research Committee, Hamadan University of Medical Sciences, Hamadan, Islamic Republic of Iran; d Research Center for Molecular Medicine, Hamadan University of Medical Sciences, Hamadan, Islamic Republic of Iran; e Department of Virology, School of Medicine, Hamadan University of Medical Sciences, Hamadan, Islamic Republic of Iran; f Department of Infectious Disease, Faculty of Medicine, Alborz University of Medical Sciences, Alborz, Islamic Republic of Iran; g Molecular Diagnosis Department, Farzan Molecular and Pathobiology Laboratory, Hamadan, Islamic Republic of Iran; h Department of Community Medicine, Hamadan University of Medical Science, Hamadan, Islamic Republic of Iran

**Keywords:** Parvovirus B19 (B19V), Parvovirus 4 (PARV4), Blood donors, Seroprevalence, Real-Time PCR, Transfusion-transmitted infections, Bloodborne pathogens, Prevalence report

## Abstract

**Background:**

Parvoviruses, characterized by their tropism for blood cells, can manifest as asymptomatic infections. With their ability to persist in blood, assessing the prevalence of Parvovirus B19 (B19V) and Parvovirus 4 (PARV4) among healthy blood donors is essential for evaluating the potential transmission risks through blood transfusions, emphasizing the need for comprehensive screening protocols.

**Methods:**

Four hundred blood donors participated in the study, with their blood specimens subjected to Real-Time PCR analysis for B19V and PARV4 nucleic acids after obtaining informed consent. Additionally, Complete Blood Count (CBC) assessments and determination of anti-B19 V-IgM and anti-B19 V-IgG antibody titers were performed using Enzyme-Linked Immunosorbent Assay (ELISA) for all collected samples.

**Results:**

The results reveal that 12 out of 400 individuals (3 %) exhibited positive results for B19V DNA, while 6 out of 400 individuals (1.5 %) tested positive for PARV4 DNA. Additionally, 8 out of 400 individuals (2 %) displayed positive results for anti-B19V IgM, and 306 out of 400 individuals (76.5 %) exhibited positive results for anti-B19 IgG. Notably, one donation from a donor presenting anti-IgM antibodies was subsequently confirmed as B19V DNA-positive through Real-Time PCR. In the analysis of CBC, a significant disparity in platelet levels was observed between B19V-positive donors, PARV4-positive donors, and B19V-negative donors.

**Conclusions:**

The study suggests that individuals at high risk, lacking detectable B19V antibodies, should undergo systematic screening and exclusion. This precaution is intended to minimize potential contamination risks within the studied cohort, despite the undefined pathogenesis and clinical implications of PARV4.

## Introduction

Human parvovirus B19 (B19V), the smallest human DNA virus, can cause a range of diseases. It is widespread, and its manifestations can vary from asymptomatic to severe across different age groups. In immunocompetent children, it can lead to transient aplastic crisis and erythema infectiosum, while in adults, it is associated with acute polyarthropathy. Other symptoms include prolonged joint pain, chronic pure red blood cell aplasia, hydrops fetalis, spontaneous abortion, and severe anemia [1], [2]. B19V is typically transmitted through respiratory droplets. However, vertical transmission from mother to fetus can occur through blood derivatives in cellular blood components [3]. This virus is significant in the context of blood and plasma product transfusions. While there is a hypothesis that infectivity decreases in large plasma pools, experiments have shown that nearly half of adult donors have low anti-B19V antibody titers [3]. Due to the low risk of transmission and infrequent severe consequences, routine B19V screening is not standard practice. Childhood infections, such as erythema infectiosum or slapped cheek syndrome, indicate previous infection and may explain the high B19V seroprevalence in blood donors [4]. The impact of asymptomatic B19V infection on blood counts remains unclear, but reports of reduction in blood counts have been noted in B19V-infected individuals, including otherwise healthy ones [5], [6].

Parvovirus 4 (PARV4), a novel member of the Parvoviridae family, was first identified in plasma in 2005 [7]. The natural history and clinical significance of this virus remain poorly understood. Some studies have associated PARV4 infection with symptoms such as an influenza-like syndrome, encephalitis, HIV disease progression, and fetal hydrops [8]. PARV4 has been detected in the blood of febrile patients, intravenous drug users, and individuals positive for hepatitis C or HIV [9]. The virus has also been found in blood donors and in blood products negative for Parvovirus B19 DNA [7], [10].

To gain new insights into the presence of B19V and PARV4 in healthy blood donors, we conducted a study to investigate blood contamination with these infections in Hamedan province. The presence of high-risk recipients for pooled plasma derivatives prompted us to determine the seroprevalence of B19V and PARV4 in healthy blood donors. This epidemiological study aims to inform decisions related to public and high-risk health.

## Material and methods

### Participants

This cross-sectional study was conducted in coordination with the Hamedan Blood Transfusion Organization in Hamedan city, Iran. The study received ethical approval from the Hamedan University of Medical Sciences (IR.UMSHA.REC.1395.554). A cohort of 400 voluntary blood donors, aged between 19 and 62 years with a mean age of 38.7 years, was recruited for this study following the provision of informed consent. Participants were selected based on their referral to the Hamedan Blood Transfusion Organization. Exclusion criteria included refusal to participate and the presence of medical conditions contraindicating blood donation.

### Serology for anti-B19V IgG and anti-B19V IgM

We quantified anti-B19V-IgG and anti-B19V IgM using an enzyme-linked immunosorbent assay (ELISA) method. ELISA kits for these determinations were sourced from Immuno-Biological Laboratories (IBL, Switzerland), and procedures followed the manufacturer's instructions.

### DNA extraction

DNA extraction from serum and plasma samples was performed according to the manufacturer's instructions using the commercial kit High Pure viral DNA/RNA (Roche, Switzerland). Extracted DNA purity was assessed based on absorbance at 260 and 280 nm wavelengths using a Nanodrop 2000 (USA). The extracted DNA was stored at − 80 °C for further analysis.

### Real-Time PCR analysis for B19V and PARV4

We employed a sensitive Real-Time PCR test to detect B19V DNA, targeting the NS1 gene according to the Genesig Advanced Kit (Genesig, United Kingdom). SYBR Green Real-Time PCR was used to detect PARV4 DNA in the plasma of blood donors. Forward (ATGGTGGGAAGAAGGTAGAATG) and reverse (GATGACACAGGTGGGTATGTAG) primers were used for PARV4 detection, with the following PCR program: initial denaturation for 5 min at 95 °C, followed by 45 cycles of 20 s at 95 °C, 30 s at 60 °C, and 10 s at 72 °C.

### Blood count

A complete blood count for each donor was performed using a Mindray BC-5150 auto hematology analyzer (Mindray 5-part blood test analyzer) with EDTA blood samples collected from sampling pouches for diversion.

### Statistical analysis

All demographic, clinical, and experimental data were analyzed using SPSS software version 20 (SPSS Inc., Chicago, IL, USA). Descriptive analysis was performed for individual characteristics. Categorical variables were expressed as percentages, and group differences were assessed using the chi-squared test, Independent Sample T-test, Kruskal-Wallis, and One Way ANOVA test. P-values of < 0.05 were considered statistically significant.

### Statistical analysis

All demographic, clinical, and experimental data were analyzed using SPSS software version 20 (SPSS Inc., Chicago, IL, USA). Individual characteristics were descriptively analyzed. Categorical variables were expressed as percentages, and differences between groups were judged for significance using the chi-squared test, Independent Sample T-test, Kruskal- Wallis, One Way ANOVA test. The p-values of < 0.05 were considered statistically significant.

## Result

### Frequency of B19V IgM and B19V IgG in blood donors

No significant differences were observed in participant ages (P > 0.05). The seropositivity rate of blood donors for B19V-IgG and B19V-IgM was 76.5 % (306/400) and 2 % (8/400), respectively (Table 1).

**Table 1:** Demographic data frequency of seropositivity of B19V IgG and B19V IgM in all blood donors.

Number	Age median (Min-Max)	Gender (Male/Female)	B19v IgG seropositivity		B19v IgM seropositivity	
			Positive	Negative	Positive	Negative
400	38.752 (19–62)	(384/16)	306(76.5 %)	94(23.5 %)	8(2 %)	392(98 %)

### Real-Time PCR results

The positivity rate for B19V-DNA in blood donors was 3 % (12/400), with 2.9 % (11/384) in males and 6.3 % (1/16) in females. PARV4-DNA was found in 1.5 % (6/400) of cases, all of which were male (Table 2).

**Table 2:** Frequency of B19V-DNA and PARV4-DNA in blood donors.

B19V DNA				PARV4 DNA			
Male		Female		Male		Female	
Positive	Negative	Positive	Negative	Positive	Negative	Positive	Negative
11(2/9 %)	373(97/1 %)	1(6/3 %)	15(93/7 %)	6(1/6 %)	378(98/4 %)	0(0 %)	16(100 %)
384		16		384		16	

### Blood count

No significant difference was observed in blood counts between B19V-IgG seropositive and seronegative blood donors (Table 3). However, B19 seropositive blood donors exhibited significant differences in neutrophil, eosinophil, MCH, MCHC, and platelet counts compared to seronegative blood donors (P < 0.05) (Table 3). Platelet count in donors positive for B19V-DNA significantly differed from PARV4-DNA-positive donors (P < 0.05) (Table 4).

**Table 3:** Hematological data of 306 samples tested positive for B19V IgG compared to 94 B19V IgG negative samples.

Blood donors	All donors tested positive for B19V IgG, mean (min.–max.)	All donors tested negative for B19V IgG, mean (min.–max.)	p value
**Number of donors**	306	94	
**WBC**	6.9(3.88–12.43)	6.92(4.93–11.55)	0.907
**Neu**	55.4(35.6–79.7)	53.26(36.7–72.4)	0.018
**Lym**	33.99(13.6–55.4)	35.52(20.4–53.7)	0.071
**Mon**	8.14(2.8–74)	8.08(3.5–20.7)	0.897
**Eos**	2.45(0.2–8.5)	2.87(0.4–11.9)	0.032
**Bas**	0.35(0–1.3)	0.37(0–1.6)	0.380
**RBC**	5.53(4.36–7.6)	5.62(4.66–7.53)	0.098
**HGB**	16.42(11.2–21.7)	16.37(13–18.8)	0.735
**HCT**	47.19(33.5–60.2)	47.4(40.8–53.9)	0.609
**MCV**	85.48(65.2–101.8)	84.59(56.1–92.5)	0.092
**MCH**	29.85(21.8–60.6)	29.21(19–32.6)	0.023
**MCHC**	34.79(31.6–36.4)	34.53(31.6–36.8)	0.003
**PLT**	245.65(107–441)	244.28(132–501)	0.029

**Table 4:** Platelet count of 12 samples tested positive for B19V-DNA compared to 6 samples tested positive for PARV4-DNA in blood donors.

Blood donors	All donors tested positive for PARV4-DNA Mean (Min.–Max.)	All donors tested positive for B19V-DNA Mean (Min.–Max.)	p value
Number of donors	6	12	-
PLT	239.83(153–326)	171.00(103–238)	0.041

## Discussion

Viral diseases have long been a matter of global concern, casting their pervasive shadow over communities worldwide and persisting as one of the fundamental challenges in public health. Viruses, as microscopic agents of disease, have captivated the attention of researchers across diverse fields, including medicine, biochemistry, and epidemiology [11], [12], [13]. B19V primarily infects infants and children and typically does not cause major health problems. However, in high-risk groups, it can lead to serious complications [14]. It is estimated that 30–75 % of the world's population is positive for B19 antibodies due to childhood infection [15]. The virus is mainly transmitted through respiratory secretions, but transfusion can also be a route of transmission. There are no subjective methods for assessing asymptomatic carriers [16].

In our study, we aimed to investigate blood contamination with B19V and PARV4 in healthy blood donors. We found that 76.5 % (306/400) of blood donors tested positive for B19V IgG, while 2 % (8/400) were positive for B19V IgM. Our findings are consistent with a study by Mahmudi et al., which reported a 0.5 % (4/730) positivity rate for B19V IgM, although all participants were negative for B19V DNA [17]. In another study, Kumar et al. reported positivity rates of 27.96 % for B19V IgG and 7.53 % for B19V IgM in blood products [18]. Similarly, Francois et al. found B19V prevalence rates of 62.2 % for IgG and 0.06 % for IgM [19]. Additionally, the prevalence of B19V-specific IgG was 55 % in Chile [20]. These differences may be attributed to varying levels of hygiene and bioprotection. Furthermore, our study showed a 3 % positivity rate for B19V DNA, with 2.9 % in males and 6.3 % in females. The detection rate of B19V DNA varies, as studies have reported rates of 0.9 % in South Africa [19], 1.1 % in Brazil [21], and 0.9 % in the United States [22]. Several factors can influence these results, including sample size, choice of primers for detection, genetic diversity, natural variation in B19V circulation, and differences in sampling time. It is worth noting that B19V prevalence has been reported throughout the year, with a peak in spring [23]. These factors can explain the variability in B19V prevalence across studies. Since B19V DNA can be detected in blood for months to years after acute infection [24], screening for B19V DNA in blood products using efficient methods like Real-Time PCR should be considered in blood transfusion organizations.

Furthermore, the detection rate of B19V IgG was 76.5 %, while that of B19V DNA was 3 %. This suggests that most B19V-infected blood donors either do not have active B19V replication or have replication levels below the detection limit.

The clinical consequences of PARV4 infection remain unclear, but reported associations include an influenza-like syndrome, encephalitis, HIV disease progression, and fetal hydrops [7]. Our study found PARV4 DNA in 1.5 % (6/400) of cases, all of which were male. Previous study has documented the occurrence of PARV4 infection among Iranian blood donors [25]. Our results align with Asiyabi et al.'s study, which reported high DNA prevalence in both HIV-infected patients (35.3 %) and healthy donors (16.6 %) [25]. However, Rosenfeldt et al. observed a low incidence of Parvovirus 4 in HIV-infected children in Denmark. In their study, four children (8.7 %) had detectable PARV4 IgG, and one patient had IgM, while PARV4 DNA was negative for all participants [26]. Additionally, Matthews et al. reported a negative correlation between PARV4 IgG and HIV status in children in South Africa [27]. Touinssi et al. reported an overall prevalence of 24.0 % for PARV4 DNA in blood donors [9]. In the USA, the PARV4 DNA detection rate in blood donors was 2 % [28]. PARV4 DNA has also been identified in liver tissue (15 %), lung (23.5 %), kidney (18 %), bone marrow (5.5 %), and skin (4 %) from non-HIV-infected individuals [29], [30].

The transmission of HIV-1, HCV, and HBV viruses through blood products has significantly decreased due to routine screening for blood-borne viruses. It may be feasible but expensive to test all blood products for emerging pathogens. While modern viral inactivation procedures may not completely eliminate PARV4 from blood products, discussions persist regarding the necessity of donation screening and enhanced measures to eradicate the virus [8]. The presence of PARV4 in blood products, particularly plasma-derived ones, poses a risk to recipients [31], [32]. Notably, PARV4 is linked to injection drug users and individuals who have received blood products, suggesting a risk of parenteral transmission [33]. Considering the above information, the cost of testing blood products for PARV4 would involve expenses related to implementing screening procedures, conducting tests, and potentially discarding contaminated products. Conversely, not testing for PARV4 may lead to the inadvertent transfusion of contaminated blood products, resulting in adverse health outcomes for recipients and potential legal liabilities for blood banks and healthcare facilities. In terms of monetary value, the cost of testing for PARV4 may vary based on the scale of testing, the sensitivity of the assays used, and the testing frequency. However, the cost of ensuring blood product safety by testing for PARV4 is likely justified by the potential risks associated with virus transmission to recipients. The economic impact of using contaminated blood products without testing could surpass the costs of implementing screening measures, considering the potential consequences of infections and associated healthcare expenses. In this approach, high-risk patients for whom B19V and PARV4 infections could pose problems would receive the safest blood products [34]. To date, the need for B19V and PARV4 nucleic acid testing in blood products remains unclear. Jia et al. stated that the positive rates of B19V and PARV4 DNA in plasma pool samples were 25.64 % and 14.10 %, respectively [35]. Additionally, Kishore and Kishore mentioned that 21.3 % of B19V-infected patients had various conditions, including multi-transfused thalassemia [36]. Moreover, Javanmard et al. reported a prevalence of 12.8 % for B19V DNA and 9.3 % for PARV4 DNA among Iranian patients with hemophilia [37]. Considering these statistics, we can infer that both B19V and PARV4 are prevalent in the population, with B19V showing a higher prevalence compared to PARV4. The rates of infection with these viruses highlight the importance of screening blood donors and implementing stringent measures to prevent transfusion-transmitted infections. These findings underscore the need for continued vigilance in transfusion medicine to minimize the risk of viral transmission through blood products. The evidence above advocates for the ongoing implementation of regular monitoring and continuous studies in this field. Routine surveillance is essential to promptly identify and address emerging threats, ensuring the safety and efficacy of blood transfusion practices. Furthermore, sustained research efforts will enhance our understanding of the epidemiology of these infections and the development of more effective prevention and treatment strategies. We will also consider factors such as the presence of anti-B19 and anti-PARV4 antibodies, their intensity and titers in blood products, the immune status of recipients, recipients' infection history, and viral persistence. We recommend screening transfusion samples for parvovirus B19V and PARV4 using IgM ELISA, complemented by sensitive nucleic acid testing methods like Real-Time PCR in high-risk patients. Technological advancements, including pathogen inactivation and nanofiltration techniques, provide additional strategies for enhancing the safety of blood products concerning B19V and PARV4. These methods effectively reduce viral particle counts in the final product, thereby mitigating the risk of transmission. Incorporating such advanced technologies into blood processing protocols not only ensures a higher safety standard but also aligns with current best practices in transfusion medicine and infection control.

## Conclusion

This study detected human B19V and PARV4 DNA in 3 % and 1.5 % of cases, respectively. Additionally, a high prevalence of anti-B19V IgG (76.5 %) was found among blood donors, which may pose a transfusion risk, particularly for high-risk patient groups. Therefore, it is recommended to consider enhancing blood and transfusion safety by screening blood units for B19V and PARV4 using IgM ELISA. Complementary methods such as Real-Time PCR, based on nucleic acid detection, are also advisable.

## Ethical approval

All procedures performed in studies involving human participants were in accordance with the ethical standards of the national research committee (Hamadan University of Medical Sciences) (IR.UMSHA.REC.1395.554) and with the 1964 Helsinki declaration and its later amendments or comparable ethical standards.

## Funding

This study did not receive any specific grant from any funding agencies in the public, commercial or not-for-profit sector.

## CRediT authorship contribution statement

**Mojtaba Hedayat Yaghoubi:** Formal analysis, Project administration. **Mohammad Masoud Maleki:** Formal analysis, Project administration. **Zahra Sanaei:** Project administration, Software. **Farid Azizi Jalilian:** Conceptualization, Methodology, Supervision, Validation, Visualization. **Mohammadmahdi Sabahi:** Conceptualization, Supervision. **Azin Kazemi:** Data curation, Formal analysis, Resources. **Razieh Amini:** Conceptualization, Supervision, Validation, Visualization. **Mehrdad Mosadegh:** Investigation, Methodology, Writing – original draft, Writing – review & editing. **Shahab Mahmoudvand:** Data curation, Investigation, Project administration, Resources.

## Declaration of Competing Interest

The authors declare that they have no known competing financial interests or personal relationships that could have appeared to influence the work reported in this paper.

## Data Availability

Authors are required to make materials, data, code, and associated protocols promptly available to readers without undue qualifications.
